# Association of trajectory of body shape index with all-cause and cause-specific mortality: 18 years follow-up

**DOI:** 10.3389/fendo.2023.1259849

**Published:** 2023-12-08

**Authors:** Elham Kazemian, Ladan Mehran, Safdar Masoumi, Atieh Amouzegar, Fereidoun Azizi

**Affiliations:** ^1^ Non-Communicable Diseases Research Center, Alborz University of Medical Sciences, Karaj, Iran; ^2^ Endocrine Research Center, Research Institute for Endocrine Sciences, Shahid Beheshti University of Medical Sciences, Tehran, Iran

**Keywords:** body mass index, body shape index (ABSI), mortality, cardiovascular disease, trajectory

## Abstract

**Objectives:**

The current study aimed to examine how the trajectory of a body shape index (ABSI) could predict mortality in a prospective cohort of 5587 participants.

**Methods:**

A Growth Mixture Model (GMM) was employed to identify ABSI and body shape trajectories spanning from 2000 to 2018. Multivariate Cox regression models with hazard ratio (HR) and 95% confidence intervals (CIs) were built to assess the association of death from all-cause and cardiovascular disease (CVD) with ABSI and body shape trajectories.

**Results:**

We found that individuals with a low ABSI–marked increase (Class II) and high ABSI–marked increase trajectory (Class III) had a higher risk of all-cause (adjusted HR for Class II, 1.37; 95%CI, 1.04-1.79; adjusted HR for Class III, 1.42; 95%CI, 1.05-1.91) and non- CVD mortality (adjusted HR for Class II, 1.38; 95%CI, 1.00-1.91; adjusted HR for Class III, 1.42; 95%CI, 1.00-2.05) as well as an increased risk of CVD (adjusted HR for Class II, 1.40; 95%CI, 1.14-1.71; adjusted HR for Class III, 1.42; 95%CI, 1.13-1.78) and coronary heart disease (CHD) (adjusted HR for Class II, 1.52; 95%CI, 1.18-1.96; adjusted HR for Class III, 1.47; 95%CI, 1.11-1.95. The trajectories of body shape phenotypes did not show any significant associations with mortality, CVD, or CHD events.

**Conclusions:**

ABSI trajectories might be associated with subsequent risk of mortality and CVD events.

## Introduction

Over the last three decades, the increased prevalence of obesity has become a public health concern in both developed and developing countries (1). Excessive body weight, as determined by body mass index (BMI) BMI, is associated with increased risks of major health problems such as diabetes, hypertension, cardiovascular disease, cancer, and subsequent mortality (2–7). However, skepticism has arisen regarding the effectiveness of BMI as a predictor for severe obesity outcomes, challenging the credibility of BMI-based obesity guidelines (8–10). First, BMI does not differentiate between fat and muscle mass, and studies indicate that elevated fat mass is a more accurate predictor of higher mortality rates compared to increased body mass in general (8). It is shown that abdominal size is positively correlated with metabolic complications of obesity, whereas gluteofemoral size shows an inverse correlation (11). However, BMI fails to assess central obesity, which is linked to a higher risk of premature mortality. To address this limitation, waist circumference (WC) was introduced as a complementary measurement to BMI, but studies showed that WC is still sensitive to weight and height and is highly correlated with BMI, which leads to collinearity (12, 13). To address these shortcomings, a body shape index (ABSI) and hip index (HI) that is independent of BMI has been developed, which uses WC and HC adjusted for height and weight. ABSI and HI, which are associated with body volume, assess transversal body dimensions (waist and hip circumference) in a manner similar to how BMI compares body mass among individuals of the same height and weight. ABSI has shown positive correlations, while HI demonstrates negative associations, with mortality, cardio-metabolic risk factors, and various malignancies, including certain cancers not traditionally associated with obesity (14–19). For example, a retrospective study conducted on 6081 adults over 18 years old demonstrated increased all-cause, CVD, and cancer-related mortality rates associated with higher ABSI values (18). In another prospective cohort study of 7414 participants aged 18 or higher, ABSI was identified as a predictor of all-cause mortality(18). As of our current knowledge, there are no published longitudinal studies that have explored the direct relationships between the trajectory patterns of ABSI and HI over specific time spans and their subsequent effects on mortality. To investigate this unexplored area, we examined how body shape trajectories, assessed through ABSI, could predict the risks of all-cause and cause-specific mortality in the prospective cohort of the Tehran Lipid and Glucose Study (TLGS) over an 18-year follow-up period. Furthermore, participants’ body shape phenotype was assessed prospectively, and they were subsequently followed until death, enabling causal inference through mapping long-term patterns.

## Methods

### Study population

The TLGS is a population-based prospective cohort study conducted from 1999 to 2002, encompassing 15,005 men and women aged ≥3 years residing in Tehran’s neighborhood No.13, serving as a representative sample of the city’s population. The TLGS participants have been followed up roughly every three years for two decades, and data has been collected in six follow-ups. The methodologies and design of TLGS have been previously documented (20, 21). Participants were invited to the TLGS unit and given written informed consent by trained social workers. Self-reported standard questionnaires were used to collect demographic and lifestyle information. Qualified physicians then interviewed the participants to get information about their medical history, smoking habits, and physical examination. Blood pressure (both systolic and diastolic) (mm Hg) was calculated by taking the average of two measurements made after a five-minute break in a regular sitting position using a standard mercury sphygmomanometer. Anthropometric measurements were obtained according to standard protocol, with the subjects wearing light clothing and their shoes removed. All study participants had their blood drawn after an overnight fast of 12–14 hours. On the day of blood collection, biochemical measurements such as fasting plasma glucose (FPG) and all blood lipid analyses were performed at the TLGS research laboratory using a Selectra 2 autoanalyzer (Vital Scientific, Spankeren, Netherlands). TLGS participants were defined as diabetic if their fasting glucose was higher than 126 mg/dL or if they were on diabetes medication.

The current study included all TLGS participants over the age of 35 who were free of cardiovascular disease and cancer at baseline and completed the baseline assessment and at least two additional follow-up re-examinations between the second (2002–2005) and sixth (2015–2018) follow- up visits (n=8352) before the event happened. Moreover, participants with pre-existing cardiovascular disease (n = 596) and cancer at the beginning of the study (n = 44), individuals lacking data on ABSI at the baseline (n = 833) and throughout all follow-up periods (n = 1292), as well as those with missing covariate information (n = 169), were excluded from the analysis. The final analysis included 5587 adults aged 35 to 80 years, with 2930 (52.44%) women and 2657 (47.56%) men; 893 (15.98%) were smokers at baseline, and 3863 (69.14%) had no education or completed primary education. We chose an age cutoff of 35 years and above based on epidemiological evidence indicating that premature coronary artery disease (CAD) in men under 45 years and women under 55 years constitutes a relatively small percentage, ranging from 3% to 10%, of the overall CAD cases (22).

### Assessment of the anthropometric measures

WC was measured at the level of the umbilicus, and hip circumference was measured at the widest girth of the hip (both in cm) (20). BMI was calculated by dividing weight (in kg) by the square of height (in m) (20). The ABSI was designed as a risk indicator, taking into account the elevated risk associated with WC while considering BMI and height as contributing factors (14). The ABSI was derived from the analysis of data collected during the 1999-2004 US National Health and Nutrition Examination Survey (NHANES), inluding non-pregnant adults aged 18 years and above. ABSI was calculated by the following formula (WC/BMI^2/3^ * height^1/2^) (14). According to the ABSI formula, a high ABSI value implies an individual has a waist circumference exceeding the expected measurements for their weight and height, indicating a more concentrated distribution of body volume in the central region. HI was calculated based on the following formula:


$$HI\;=\;HC\;\left(cm\right)\times \left(\;Weight\;{\left(kg\right)}^{-0.482}\;\times Height{\left(cm\right)}^{0.310}\right)$$

For the current analysis, ABSI and HI were dichotomized using rounded numbers close to the sex-specific medians in the complete study dataset at enrolment: ABSI: 0.58 for women and 0.60 for men, HI: 49 for women and 64 for men. Body shapes were categorized as “pear” (low-ABSI-high-HI), “apple” (high–ABSI–low–HI), “slim” (low–ABSI–low–HI), and “wide” (high–ABSI–high–HI) phenotypes. The ABSI and body shape phenotypes were calculated from anthropometric measurements at six follow-up periods of 1999–2001, 2002–2005, 2006–2008, 2009–2011, 2012–2014, and 2015–2018.

### Outcomes

In this prospective analysis, the primary outcomes were overall and cause-specific mortality, including death from cardiovascular disease (CVD), non-CVD causes, and non-cancer causes. A cardiovascular event was defined as any confirmed myocardial infarction (MI), probable MI, unstable angina pectoris, angiographically proven coronary artery disease, congestive heart failure, sudden cardiac death, fatal or non-fatal stroke, as well as deaths attributed to cardiovascular disease (CVD) events (23, 24). TLGS participants are contacted annually by phone to inquire about any medical events, such as CVDs and deaths, occurring in the previous year. All events are adjudicated by an outcome committee composed of an internist, endocrinologist, pathologist and cardiologist, and epidemiologist. Deaths are verified by death certificates. Comprehensive investigations are undertaken to ascertain the cause of death, involving a thorough review of the death certificate, all medical records, and any information provided by attending physicians, medical examiners, and family members.

### Statistical analysis

#### Latent trajectory classes of ABSI

The ABSI trajectory was determined using a latent class trajectory analysis model implemented with the ‘lcmm’ package in R software. This model assumes that each participant belongs to one of several latent classes, and repeated measures of participants in the same latent class followed a linear mixed-effects model. The number of latent classes and their class size in the study population were estimated from the data, and the most optimal path shape and number of classes were identified using Bayesian information criterion, high posterior probability (0.7), mean probability of group membership, and root mean error of approximation (RMEA). ABSI was considered a dependent variable, and time was expressed in years according to the age of the participant at each follow-up visit, while gender was controlled as a confounding factor. The number of route groups was limited to a maximum of five groups. To ensure that all subsequent classes were clinically meaningful in size, we enforced the requirement that each class included at least 5% of participants and discarded results from models with less than 5% of participants. Participants with missing data at baseline were excluded. The rate of data lost to follow-up in the TLGS was less than 5%, and data were lost at random, so complete case analysis was applied.

#### Trajectory associations of ABSI and all-cause and cause-specific mortality

Descriptive statistics were presented according to the ABSI trajectory groups. Differences by ABSI trajectory groups were explored by one-way analysis of variance, Kruskal Wallis H test, and chi-square tests. Multivariate Cox regression models with hazard ratios (HRs) and 95% confidence intervals (CIs) were built to assess deaths from all-cause and CVD. All models were adjusted for the mean age of follow-up, sex, smoking status, family history of CVD, physical activity, lipid drugs score, and hypertension drugs score. Participants’ usage of antihypertensive and lipid-lowering drugs was assessed, with scores ranging from 0 to 6 (0 indicating no usage and 6 indicating usage during all six follow-up visits). The models were additionally adjusted for the mean follow-up values of FPG, SBP, as well as TC and triglycerides (TG). Kaplan-Meier curves and log-rank tests were employed to examine differences in survival rates across ABSI trajectory groups. Results from Cox regression were stratified by never-smokers and non-diabetics at baseline. Statistical analyses were conducted using STATA version 14.2 and R software, with statistical significance determined at a threshold of P < 0.05.

## Results

**Figure 1:**
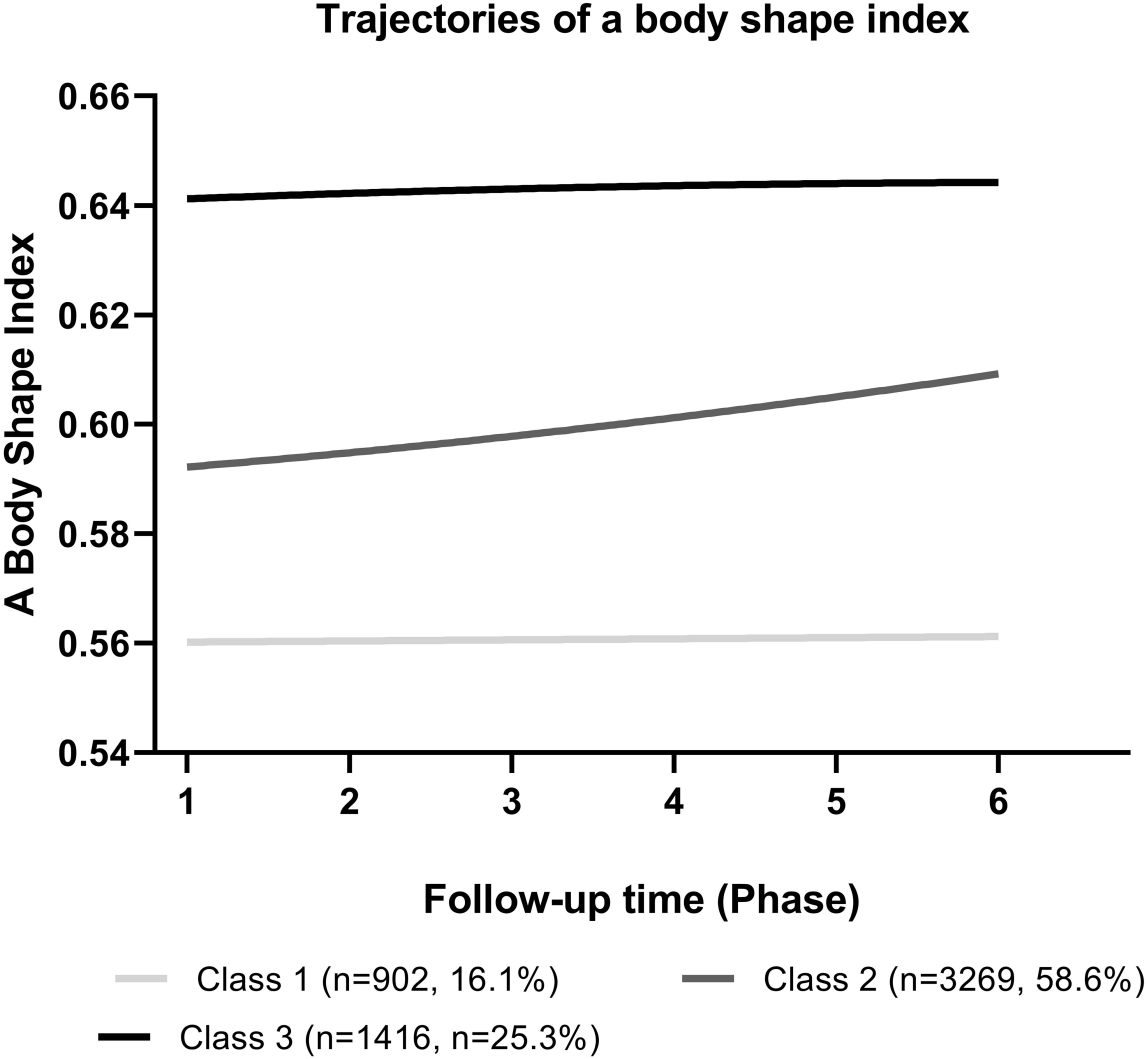
The Body Shape Index (ABSI) trends in different trajectory classes among Tehran Lipid Glucose Study participants (2001-2018).

Analyses were conducted on a total of 5587 adults. Over a 15.8-year average follow-up period from 2001 to 2018, 566 deaths from all causes (178 from CVD and 388 non-CVD) and 1024 CVD events were recorded. At baseline, the study involved individuals with an average age of 48.62 years (SD 10.35), with 2930 (52.44%) being women and 2657 (47.56%) men respectively. Based on both baseline ABSI and trends over time, three distinct trajectory patterns were identified (**Figure 1**): Class 1: Low ABSI–moderate increase (n = 902, 16.1%), Class II: Low ABSI–marked increase (n = 3269, 58.6%), Class III: High ABSI–marked increase (n = 1416, 25.3%).

The basic characteristics of study participants at baseline, according to ABSI trajectories from 1999-2001, are depicted in **Table 1**. There were significant differences in BMI, SBP, DBP, FBS, TC, and TG across different ABSI trajectory groups (P<0.05).

**Table 1:** Characteristics of participants by a body shape index trajectory groups in the Tehran Lipid and Glucose Study.

Characteristic	Total	ABSI trajectory groups			P-value
		Class I	Class II	Class III	
**NO.**	5,587	902	3,269	1,416	
Age, years*	48.62±10.35	48.82±10.42	49.08±10.39	47.42±10.12	**<0.001**
**Female^+^**	2930 (52.44)	731 (81.04)	1622 (49.62)	577 (40.75)	**<0.001**
**Education level^+^**					0.49
Illiterate/primary	3863 (69.14)	636 (70.51)	2264 (69.26)	963 (68.01)	
Secondary/diploma	1090 (19.51)	178 (19.73)	631 (19.30)	281 (19.84)	
≥12 years	634 (11.35)	88 (9.76)	374 (11.44)	172 (12.15)	
Current smoking^+^					<0.001
Yes	893 (15.98)	68 (7.54)	532 (16.27)	293 (20.69)	
No	4694 (84.02)	834 (92.46)	2737 (83.73)	1123 (79.31)	
**Low Physical activity^+^**	3820 (68.37)	583 (64.42)	2304 (70.24)	933 (66.55)	**0.001**
**BMI (kg/m^2^ )**	27.69±4.53	30.19±5.25	27.83±4.28	25.76±3.67	**<0.001**
**BMI categories** (**kg/m2)^+^**					**<0.001**
BMI<30	4058 (72.63)	465 (51.55)	2348 (71.83)	1245 (87.92)	
BMI≥30	1529 (27.37)	437 (48.45)	921 (28.17)	171 (12.08)	
SBP, mm Hg*	122.13±19.43	123.66±19.90	122.43±19.14	120.44±19.69	**0.001**
DBP, mm Hg*	79.26±11.06	80.10±10.91	79.44±11.16	78.29±10.84	**0.001**
FBS (mg/dL)*	101.77±35.75	97.20±26.39	102.05±36.00	104.06±39.90	**<0.001**
TC (mg/dL)*	215.75±45.52	218.52±46.34	215.93±44.99	213.57±46.16	**0.036**
TG (mg/dL)*	187.93±126.8	169.27±93.42	189.84±132.54	195.50±130.80	**<0.001**
**Family history CVD^+^**	918 (16.43)	172 (19.01)	512 (15.61)	234 (16.69)	**0.049**
**All-cause mortality^+^**	566 (10.13)	64 (7.10)	364 (11.13)	138 (9.75)	**0.002**
**CVD mortality^+^**	178 (3.19)	18 (2.0)	116 (3.55)	44 (3.11)	**0.062**
**CVD event^+^**	1024 (18.33)	114 (12.64)	642 (19.64)	268 (18.93)	**<0.001**

BMI, Body mass index; SBP, Systolic blood pressure; DBP, Diastolic blood pressure; FBS, Fasting blood sugar; TC, Total cholesterol; TG Triglycerides, CVD; Cardiovascular disease.

Class I: Low ABSI – moderate increase; Class II: Low ABSI – marked increase; Class III: High ABSI – marked increase.

^*^mean±SD; ^+^No(%).

HRs for all-cause, CVD, non-CVD, and non-cancer mortality based on ABSI trajectory groups are presented in **Table 2**, stratified by smoking and diabetes status. Compared to the low ABSI–moderate increase trajectory (Class 1), both the low ABSI–marked increase (Class II) (adjusted HR, 1.37; 95%CI, 1.04-1.79) and high ABSI–marked increase (Class III) trajectory (adjusted HR, 1.42; 95%CI, 1.05-1.92) were associated with a higher subsequent risk of all-cause mortality as well as non-CVD mortality (adjusted HR for Class II, 1.38; 95%CI, 1.00-1.91; adjusted HR for Class III, 1.42; 95%CI, 1.00-2.05), after accounting for potential confounders.

**Table 2:** HRs for mortality according to body shape index trajectory groups in the Tehran Lipid and Glucose Study.

	Overall*			Never-smokers†			Non-diabetic‡		
	Death (%)	HR (95 % CI)	P-value	Death (%)	HR (95 % CI)	P-value	Death (%)	HR (95 % CI)	P-value
All-cause mortality									
Class I	64 (7.10)	1.0 (RE)	–	57 (6.82)	1.0 (RE)		53 (6.30)	1.0 (RE)	–
Class II	364(11.13)	**1.37 (1.04-1.79)**	**0.02**	293 (10.70)	**1.33(0.99-1.78)**	**0.051**	262 (8.91)	**1.39(1.03-1.88)**	**0.03**
Class III	138(9.75)	**1.42 (1.05-1.92)**	**0.02**	106(9.44)	**1.40(1.00-1.94)**	**0.044**	100 (8.06)	**1.43(1.01-2.01)**	**0.04**
CVD mortality									
Class I	18 (1.99)	1.0 (RE)	–	18 (2.16)	1.0 (RE)	–	15 (1.78)	1.0 (RE)	–
Class II	116 (3.55)	1.36 (0.81-2.27)	0.23	83 ( 3.03)	1.37(0.82-2.29)	0.22	78 (2.65)	1.20(0.68-2.11)	0.52
Class III	44 (3.11)	1.32 (0.74-2.32)	0.33	26(2.32)	1.4(0.80-2.47)	0.23	29 ( 2.34)	1.11(0.58-2.10)	0.74
Non-CVD mortality									
Class I	46 (5.19)	1.0 (RE)	–	39 (4.77)	1.0 (RE)	–	38 (4.60)	1.0 (RE)	–
Class II	248 (7.87)	**1.38(1.00-1.91)**	**0.048**	210(7.91)	**1.44(1.01-2.04)**	**0.042**	184 (6.43)	**1.46(1.02-2.10)**	**0.037**
Class III	94 (6.85)	**1.42(1.00-2.05)**	**0.052**	80 (7.29)	**1.54(1.04-2.27)**	**0.031**	71 (5.86)	**1.52(1.02,2.28)**	**0.040**
Non-cancer mortality									
Class I	57 (6.37)	1.0 (RE)	–	51 (6.14)	1.0 (RE)	–	46 (5.52)	1.0 (RE)	–
Class II	303 (9.45)	1.29(0.97-1.73)	0.085	237 (8.84)	1.23(0.90-1.67)	0.19	210 (7.27)	1.31(0.94-1.82)	0.099
Class III	128(9.10)	**1.46(1.06-2.01)**	**0.021**	96 (8.63)	**1.43(1.00-2.02)**	**0.042**	90 (7.31)	**1.48(1.03-2.13)**	**0.035**

DBP, Diastolic blood pressure; FBS, Fasting blood sugar; TC, Total cholesterol; CVD, Cardiovascular disease; HR; hazard ratio.

Class I: Low ABSI – moderate increase; Class II: Low ABSI – marked increase; Class III: High ABSI – marked increase.

*Adjusted for mean age at follow-up, sex, smoking status, family history of CVD, physical activity, lipid drug and hypertension drug score as well as mean follow-up values for FPG, SBP, TC, and TG.

† Adjusted for mean age at follow-up, sex, family history of CVD, physical activity, lipid drug score, hypertension drug score as well as mean follow-up values for FPG, SBP, TC, and TG.

‡ Adjusted for mean age at follow-up, sex, smoking status, family history of CVD, physical activity, lipid drug and hypertension drug score as well as mean follow-up values for SBP, TC and TG. Bold values denote statistical significance at the p < 0.05 level.

Furthermore, when cancer-related deaths were excluded from the analysis, the high ABSI– marked increase trajectory remained associated with a higher mortality risk compared to the low ABSI–moderate increase trajectory (adjusted HR, 1.46; 95%CI, 1.06-2.01). The association between ABSI trajectories and the risk of non-CVD mortality appeared stronger in individuals who never smoked and those without diabetes.

**Figure 2:**
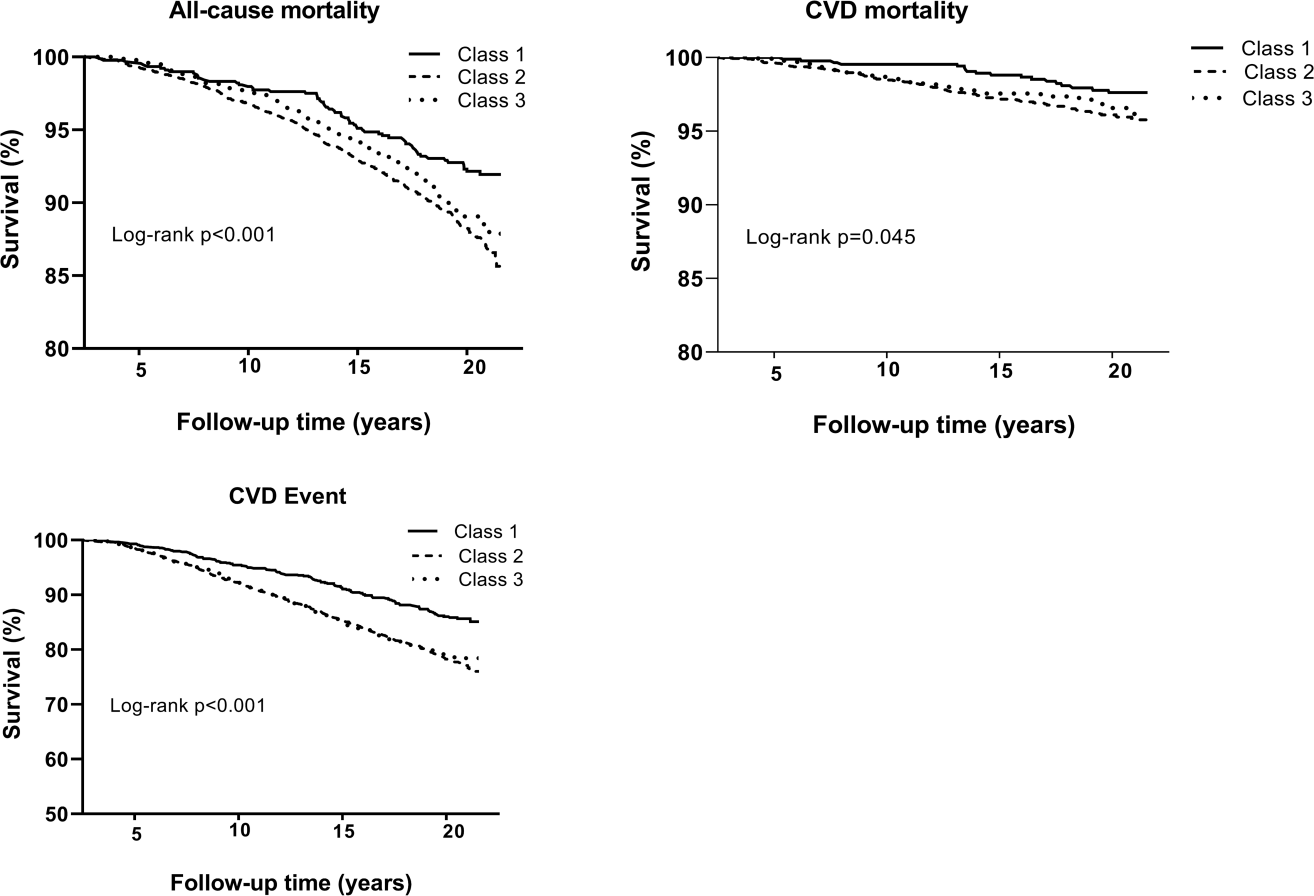
(A) Kaplan-Meier survival analysis for all-cause mortality, cardiovascular (CVD) disease mortality and incidence of CVD according to a body shape index trajectory.

The ABSI trajectories were also significantly associated with a higher risk of CVD and CHD incidence (**Table 3**). The low ABSI–marked increase and the high ABSI–marked increase trajectory were associated with higher subsequent risk of CVD (adjusted HR for Class II, 1.40; 95%CI, 1.14-1.71; adjusted HR for Class III, 1.42; 95%CI, 1.13-1.78) and CHD (adjusted HR for Class II, 1.52; 95%CI, 1.18-1.96; adjusted HR for CLASS III, 1.47; 95%CI, 1.11-1.95), after adjusting for potential confounders. Excluding smokers and diabetes patients did not substantially change the results. No significant interaction was observed between ABSI trajectories and sex. **Figure 2** displays Kaplan-Meier survival analyses for all-cause mortality, CVD mortality, and incidence of CVD based on ABSI trajectory.

**Table 3:** HRs for the incidence of cardiovascular disease events according to body shape index trajectory groups in the Tehran Lipid and Glucose Study Study.

	Overall*			Never-smokers†			Non-diabetic‡		
	Event (%)	HR (95 % CI)	P-value	Event (%)	HR (95 % CI)	P-value	Event (%)	HR (95 % CI)	P-value
CVD Event									
Class I	114 (12.60)	1.0 (RE)	–	106 (12.7)	1.0 (RE)	–	98 (11.65)	1.0 (RE)	–
Class II	642 (19.64)	**1.40(1.14-1.71)**	**0.001**	512 (18.71)	**1.40(1.14-1.72)**	**0.001**	500 (17.01)	**1.40(1.12-1.75)**	**0.003**
Class III	268 (18.93)	**1.42(1.13-1.78)**	**0.002**	197(17.54)	**1.45(1.16-1.82)**	**0.001**	200 (16.12)	**1.43(1.11-1.84)**	**0.005**
CHD Event									
Class I	73 (8.09)	1.0 (RE)	–	66 (7.91)	1.0 (RE)	–	62 (7.37)	1.0 (RE)	–
Class II	459 (14.04)	**1.52(1.18-1.96)**	**0.001**	364 (13.30)	**1.52(1.18-1.97)**	**0.001**	357 (12.15)	**1.55(1.17-2.04)**	**0.002**
Class III	182 (12.85)	**1.47(1.11-1.95)**	**0.007**	139 (12.38)	**1.50(1.13-2.00)**	**0.004**	137 (11.04)	**1.52(1.11-2.08)**	**0.008**
Non-CHD Event									
Class I	41 (4.95)	1.0 (RE)	–	1( 1.61)	1.0 (RE)	–	36 (4.62)	1.0 (RE)	–
Class II	183 (6.51)	1.24(0.87-1.75)	0.22	35 (8.01)	1.24(0.88-1.76)	0.21	143 (5.54)	1.21(0.83-1.77)	0.30
Class III	86 ( 6.97)	1.40(0.95-2.06)	0.08	28 (11.20)	1.44(0.98-2.11)	0.06	63 (5.71)	1.32(0.86-2.03)	0.19

HR, hazard ratio; DBP, Diastolic blood pressure; FBS, Fasting blood sugar; TC, Total cholesterol; CVD, Cardiovascular disease; CHD, Coronary heart disease.

Class I: Low ABSI – moderate increase; Class II: Low ABSI – marked increase; Class III: High ABSI – marked increase.

*Adjusted for mean age at follow-up, sex, smoking status, family history of CVD, physical activity, lipid drug and hypertension drug score as well as mean follow-up values for FPG, SBP, TC and TG.

† Adjusted for mean age at follow-up, sex, family history of CVD, physical activity, lipid drug and hypertension drug score as well as mean follow-up values for FPG, SBP, TC and TG.

‡ Adjusted for mean age at follow-up, sex, smoking status, family history CVD, physical activity, lipid drug and hypertension drug score as well as mean follow-up values for SBP, TC and TG. Bold values denote statistical significance at the p < 0.05 level.

The body shape phenotype trajectory showed no significant association with mortality, CVD, or CHD events. Stratifying the data by gender or BMI did not alter this association.

## Discussion

In this prospective study, we identified three distinct ABSI trajectories that were associated with altered mortality and CVDrisk. Among TLGS participants, a significant association was found between the increasing ABSI trend and subsequent mortality and cardiovascular disease (CVD) events, regardless of their ABSI status at baseline. Participants with either low or high ABSI at baseline and a marked increase in ABSI during follow-up experienced a 37% and 42% higher risk of all-cause mortality, respectively, and a 30-35% higher risk for CVD events compared to those with low ABSI and a moderate increase in ABSI trajectory. However, the body shape phenotype identified in this study did not influence the risk of mortality, CVD, or CHD events.

Understanding the relationship between a higher mortality rate and obesity during different life stages, including childhood, adulthood, or changes in body shape during childhood or adulthood, has posed significant challenges in research (25–28). A recent study, employing the highest BMI achieved over an individual’s lifetime as a relevant exposure measure, demonstrated that maximum BMIs within the overweight, obese I (30.0 to 34.9 kg/m²), and obese II (≥ 35.0 kg/m²) categories were associated with increased mortality rates (29). Nevertheless, a dynamic evaluation of weight status, considering changes over time rather than a static measure, has been shown to be a more accurate predictor of mortality (30). Another methodological concern in studies examining the relationship between obesity and mortality is the potential impact of reverse causation from pre-existing conditions, such as chronic diseases or undetected cancer. These conditions can lead to weight loss while simultaneously increasing the likelihood of death in individuals. Failing to address this issue could weaken or even create a false inverse relationship between body weight and mortality. The trajectory approach addresses the limitations associated with examining adiposity at specific time points by allowing repeated measurements and considering the impact of different developmental paths in body shape. In the US Health and Retirement Study (HRS), focusing on older adults aged over 50 years, individuals with upward trajectories in obese Class I (BMI 30-34.9 kg/m²) and Class II/III (BMI ≥ 35 kg/m²) experienced a 30% and 147% increased mortality risk, respectively, compared to those with stable overweight trajectories (30). The developers of the ABSI measure for abdominal obesity argued that it offered superior mortality prediction compared to traditional standards like waist circumference (WC), waist-hip ratio (WHR), and waist-height ratio (WHtR) (14). In the Rotterdam Study, a population-based research involving 2,626 men and 3,740 women aged over 55, ABSI showed a robust association with total cardiovascular and cancer mortality over a 22-year follow-up period, outperforming other anthropometric measures like BMI, waist circumference (WC), waist-height ratio (WHtR), and waist-hip ratio (WHR) (31). Additionally, ABSI was demonstrated to exhibit a stronger association with all-cause mortality than BMI, waist circumference (WC), and waist-to-height ratio (WHtR) in a prospective TLGS cohort over a 10-year follow-up period (32). In this study, we expanded upon these results by presenting comprehensive data on both all-cause and cause-specific mortality. This was achieved through a longer follow-up duration and the implementation of a trajectory approach, allowing us to identify distinct subgroups of participants with similar body shape progressions over time.

Our study results are consistent with the National Health and Nutrition Examination Survey (NHANES) involving 14,105 non-pregnant adults aged ≥ 18 years, which showed the association of ABSI with premature mortality in the general population over a five-year period (17). It is important to note that ABSI was measured only at the baseline in the NHANES study. Similarly, another prospective cohort study conducted in the British Health and Lifestyle Survey (HALS) over a 24-year follow-up period demonstrated ABSI as a strong predictor of mortality hazard compared to BMI and WHR. Notably, changes in ABSI over time were predictive of mortality risk, with higher ABSI linked to an increased risk of mortality in the HALS study (17). However, it’s worth noting that the evaluation of ABSI changes was restricted to the two HALS examinations separated by a seven-year interval. No other studies investigated the influence of ABSI trajectories on mortality and CVD over an extended follow-up period involving at least three to six subsequent examinations, as was done in our study.

A high ABSI value might indicate a larger proportion of visceral (abdominal) fat compared to peripheral tissue, considering a specific height and weight (14). Furthermore, individuals with high ABSI values tend to have a lower proportion of muscle mass in their limbs, a factor strongly correlated with mortality risks (12). Subcutaneous fat differs from visceral fat in some ways, including insulin sensitivity, lipolysis activity, and adipocytokines production, all of which contribute to the development of cardiovascular disease (33).

Studies have shown that among individuals with the same height and weight, those with a pear-shaped body (low–ABSI–high–HI) had the lowest visceral adipose tissue (VAT), while apple-shaped individuals (high–ABSI–low–HI) had the highest VAT. Conversely, slim individuals (low–ABSI–low–HI) had the lowest abdominal subcutaneous adipose tissue (ASAT), while wider individuals (high–ABSI–high–HI) had the highest ASAT (34). Although our study did not show a statistically significant relation between the trajectory of body shape phenotype and all-cause or cause-specific mortality, as well as CVD and CHD events, the notable magnitude and direction of these associations hold significant clinical implications. The absence of statistical significance might be attributed to the relatively small sample size in our study, which potentially limited the statistical power to detect significant results despite their clinical relevance (35).

This study’s strengths include its prospective design, involving a large, population-based cohort of both genders, the long follow-up period, accurate and valid data on risk factors, controlling for a wide array of potential confounders, and ongoing surveillance of mortality events based on standard criteria. Additionally, our study utilized the rigorous follow-up procedures of a well-established cohort, enabling the investigation of a diverse array of causes of death beyond just overall mortality. By integrating exposure data collected over the 18 years of follow-up, the trajectory technique employed in this study offers an appealing alternative to standard analysis. Moreover, this approach can capture the lifelong progression of body shape and categorize individuals into unique, non-overlapping groups Our study also employed multiple measurements collected over time, rather than relying on a single measurement at a specific life stage. Consequently, the trajectory approach, as opposed to studying early and late-life adiposity separately, focuses on the consistent changes in body shape over time. A dynamic assessment of weight status over time, rather than a static measurement, has been proven to be a more accurate predictor of mortality (30). The inclusion of body shape phenotype provides crucial clinical insights, especially concerning CVD risk factors. Individuals with an android or “apple” shape are more strongly associated with obesity-related health issues compared to those with a gynoid or “pear” shape (34). Furthermore, our study utilized biomedical assessments of health-related risk factors, including blood pressure, cholesterol levels, triglycerides, and FPG.

The study also has several limitations that should be mentioned. Firstly, a limited number of trajectory patterns were created, possibly not fully representing individual body shape profiles. In addition, we were unable to find a heavy stable trajectory that may be due to the limited sample size for the group of interest. In addition, the results of our study cannot easily be applied to other populations because of the Persian heritage of the study group.

In conclusion, ABSI, previously identified as a substantial risk factor for death in a US population sample (NHANES 1999–2004), displayed similar associations with mortality risk in an Iranian population sample (TLGS). ABSI trajectories are correlated with an elevated risk of death within the urban Iranian population. Prior studies have demonstrated that weight loss through diet and exercise can lead to a greater reduction in waist circumference compared to overall weight loss, resulting in a decrease in ABSI (36–38). Therefore, it’s worth examining whether mortality reductions reported in some studies (39, 40) for individuals wanting to lose weight regardless of weight change can be linked to a decrease in ABSI. Future studies will determine whether changes in ABSI can serve as a distinctive and clinically valuable biomarker for lifestyle adjustments.

## Data availability statement

The raw data supporting the conclusions of this article will be made available by the authors, without undue reservation.

## Ethics statement

The studies involving humans were approved by The Research Institute for Endocrine Sciences at Shahid Beheshti University of Medical. The studies were conducted in accordance with the local legislation and institutional requirements. The participants provided their written informed consent to participate in this study.

## Author contributions

EK: Conceptualization, Writing – original draft. LM: Writing – review & editing. SM: Formal Analysis, Writing – review & editing. AA: Conceptualization, Methodology, Writing – review & editing. FA: Conceptualization, Methodology, Writing – review & editing.
